# Catalogue of Officers and Members

**Published:** 1842-09

**Authors:** 


### CATALOGUE OF OFFICERS AND MEMBERS.

Dr. Brown the Recording Secretary, has published a card containing a complete catalogue of all the home members of the American Society of Dental Surgeons, which will be sold to the profession for distribution, at $1 50 per hundred.

Orders directed to him will be promptly filled, if accompanied with cash.

It is only by private conveyance that these cards can be transmitted to the members at a moderate expense.

The following is a complete list of the names of the officers and members of the society.

| | |
|---|---|
| H. H. HAYDEN, M. D. | PRESIDENT. |
| ELEAZAR PARMLY, M. D. | 1ST VICE-PRESIDENT. |
| LEWIS ROPER, M. D. | 2D VICE-PRESIDENT. |
| N. C. KEEP, M. D. | 3D VICE PRESIDENT. |
| SOLYMAN BROWN, M. D. | RECORDING SECRETARY. |
| CHAPIN A. HARRIS, M. D. | CORRESPONDING SECRETARY. |
| LEONARD MACKALL, M. D. | TREASURER. |
| J. H. FOSTER, M. D. | LIBRARIAN. |

*Executive Committee.*

| | |
|---|---|
| EDWARD MAYNARD, M. D. | E. TOWNSEND. |
| L. S. PARMLY, M. D. | EDWARD TAYLOR, M. D. |

P. HOUSTON, M. D.

*Examining Committee.*

H. M. HAYDEN, M. D. C. A. HARRIS. M. D.

JAMES S. GUNNELL, M. D. J. McILHENNEY.

J. SMITH DODGE, D. D. S.

*Publishing Committee.*

HUDSON S. BURR, M. D. ELISHA BAKER, M. D.

SOLYMAN BROWN. M. D. S. M. SHEPHERD,

S. W. PARMLY.

*Boston Examining and Recommending Committee.*

J. TUCKER, M. D. N. C. KEEP, M. D. J. E. FISK, M. D.

*New York Examining and Recommending Committee.*

ELEAZAR PARMLY, M. D. SOLYMAN BROWN, M. D. ELISHA BAKER, M.D.

*Philadelphia Examining and Recommending Committee.*

LEWIS ROPER, M. D. HUDSON S. BURR, M. D. S. DILLINGHAM.

*Baltimore Examining and Recommending Committee.*

C. A. HARRIS, M. D. LEON. MACKALL, M. D. ENOCH NOYES, M. D.

| | |
|---|---|
| Alcock, James, | New York. |
| Allen, C. C., M. D. | New York. |
| Allen, John, | Cincinnati. |
| Arnold Wm., M. D. | New York. |
| Arthur, R., D.D.S. | Baltimore. |
| Bacon, E., M D. | Portland, Me. |
| Baker, Charles O. | Richmond, Va. |
| Baldwin, J. C. | Newark, N. J. |
| Blake, Elihu, | New York. |
| Blakesly, Dr. | Utica, N. Y. |
| Blanding, S., M.D. | Columbia, S.C. |
| Brewster, G. G., M.D. | Portsmouth. |
| Bridges, M. K. | Brooklyn. |
| Brown, A. W. | New York. |
| Brown, B. B., M. D. | St. Louis, Mo. |
| Burr, W. H. | Philadelphia. |
| Chewning, F.B., M.D. | Richmond. |
| Clark, F. H. | New York. |
| Cleveland, J. A. | Charleston, S. C. |
| Clute, Nicholas, | Louisville, Ky. |
| Cowan, J. W. | Dover, N. H. |
| Crocker, Fred. | Sagharbour, L. I. |
| Cushman, C. T. | Columbus, Ga. |
| Cuyler, V., M. D. | Hartford, Conn. |
| Dodge, Nehemiah, | D. D. S. N. Y. |
| Dunning, E. J., M. D. | Ithaca, N. Y. |
| Dwinelle, W. H. | Cazenovia, N. Y. |
| Elliot, Wm. H. | Plattsburg, N. Y. |
| Fenn, H. N., M. D. | Rochester. |
| Fisher, N. A., M. D. | Providence. |
| Foster, C. B. | Philadelphia. |
| Foster, Dr. | Utica, N. Y. |
| Frink, J. N., M. D. | Portland, Me. |
| Grant, C. W., M. D. | Newburg, N. Y. |
| Greenwood, I. I., M. D. | New York. |
| Greenwood, W. Pitt, D.D.S. | Boston. |
| Goddard, W. H. | Louisville, Ky. |
| Hamlin, T. B. | Wythe Co. Va. |
| Harris, J., M. D. | Georgetown, Ky. |
| Hawes, A. C., M. D. | Providence. |
| Hawes, G. E. | New York. |
| Hayden, Chester, D.D.S. | Washington. |
| Hill, Amos | Norwalk, Conn. |
| Holmes, O. | Baltimore. |
| Hudson, E., M. D. | Philadelphia. |
| Hullihen, S. P. | Wheeling, Va. |
| Ide, W. E., M. D. | Zanesville, O. |
| Johnson, W., M. D. | Hagarstown. |

| | | | |
|---|---|---|---|
| Keep, Solomon, M. D. | Boston. | Rodrigues, B. A., M. D. | Charleston. |
| Kelly, E. G., M. D. | Newburyport. | Rogers, M., M. D. | Cincinnati, O. |
| Laird, O. P., M.D. | Columbus, Ga. | Ross, J. B. | Frankfort, Ky. |
| Laird, W. O. | Athens, Ga. | Rosseter, David, | New York. |
| Lathrop, W. B. | Brooklyn, N. Y. | Rowell, C. S. | New York. |
| Lord, Benjamin, | New York. | Scott, W. R., D. D. S. | Raleigh. |
| Lord, William G. | Newark, N. J. | Shepherd, J. W. | Petersburg, Va. |
| Lovejoy, John, | New York. | Smith, G. W. | New Orleans. |
| Mackall, R. C., D. D. S. | Baltimore. | Smith, J. W. | Amherst, Mass. |
| Macklin, Dr. | Vicksburg, Miss. | Somerby, R. | Louisville, Ky. |
| M'Cabe, J. C. | Petersburg, Va. | Spooner, S., M. D. | New York. |
| M'Cabe, J. D. | Richmond, Va. | Stockton, S. W. | Philadelphia. |
| M'Connell, Dr. | Richmond, Va. | Straw, S. B. | Bangor, Me. |
| M'Donald, G., M. D. | Macon, Ga. | Strickland, Benjamin, | Cleveland, O. |
| M'Grath, R., M. D. | Philadelphia. | Sumner, Dr. | Providence, R. I. |
| Merritt, Charles, | Bridgeport, Conn. | Taylor, Joseph, | Maysville, Ky. |
| Monafeldt, B. | Charleston, S. C. | Taylor, J., M.D. | Crawfordsville, Ia. |
| Morrow, D., M. D. | Tuscaloosa. | Thackston, W.W.H., D.D.S. | Farmville, Va. |
| Murrill, L. | Petersburg, Va. | | |
| Nelson, Alexander, M. D. | Albany. | Tucker, E. G., M. D. | Boston. |
| Nelson, Robert, | Albany. | Van Namee, S. | Red Hook, N. Y. |
| Parmly, D. R. | Mobile. | Ward, W. A. | Pittsburg, Pa. |
| Parmly, G. W. | New Orleans. | Ware, William, | Wilmington, N.C. |
| Parmly, Jahial, D.D.S. | New York | Wayte, John G. | Richmond, Va. |
| Parmly, Jahial, | Savannah. | Westcott, A., M. D. | Syracuse. |
| Parmly, L., D. D. S. | Mobile. | Williams, P. N. St. J., | Porto Rico. |
| Peabody N., M. D. | Boston. | Young, H. H., M. D. | Troy, N. Y. |
| Peak, J. M. | Cooperstown, N. Y. | | |

